# Wild Birds in Live Birds Markets: Potential Reservoirs of Enzootic Avian Influenza Viruses and Antimicrobial Resistant *Enterobacteriaceae* in Northern Egypt

**DOI:** 10.3390/pathogens9030196

**Published:** 2020-03-06

**Authors:** Nehal M. Nabil, Ahmed M. Erfan, Maram M. Tawakol, Naglaa M. Haggag, Mahmoud M. Naguib, Ahmed Samy

**Affiliations:** 1 Reference Laboratory for Veterinary Quality Control on Poultry Production, Animal Health Research Institute, Agricultural Research Center, Dokki, Giza 12618, Egypt; nehal_nabil84@yahoo.com (N.M.N.); ahmed.erfan10000@gmail.com (A.M.E.); maram_salah82@hotmail.com (M.M.T.); naglaahagagahri@gmail.com (N.M.H.); mahmoud.naguib@imbim.uu.se (M.M.N.); 2 Zoonosis Science Center, Department of Medical Biochemistry and Microbiology, Uppsala University, Uppsala SE-75185, Sweden; 3 Immunogenetics, The Pirbright Institute, Surrey GU24 0NF, UK

**Keywords:** Live bird markets, wild birds, influenza, critically important antimicrobials, Enterobacteriacea, Northern Shoveler

## Abstract

Wild migratory birds are often implicated in the introduction, maintenance, and global dissemination of different pathogens, such as influenza A viruses (IAV) and antimicrobial-resistant (AMR) bacteria. Trapping of migratory birds during their resting periods at the northern coast of Egypt is a common and ancient practice performed mainly for selling in live bird markets (LBM). In the present study, samples were collected from 148 wild birds, representing 14 species, which were being offered for sale in LBM. All birds were tested for the presence of AIV and *enterobacteriaceae*. Ten samples collected from Northern Shoveler birds (*Spatula clypeata*) were positive for IAV and PCR sub-typing and pan HA/NA sequencing assays detected H5N8, H9N2, and H6N2 viruses in four, four, and one birds, respectively. Sequencing of the full haemagglutinin (HA) gene revealed a high similarity with currently circulating IAV in Egypt. From all the birds, *E. coli* was recovered from 37.2% and Salmonella from 20.2%, with 66–96% and 23–43% isolates being resistant to at least one of seven selected critically important antimicrobials (CIA), respectively. The presence of enzootic IAV and the wide prevalence of AMR *enterobacteriaceae* in wild birds highlight the potential role of LBM in the spread of different pathogens from and to wild birds. Continued surveillance of both AIV and antimicrobial-resistant *enterobacteriaceae* in wild birds’ habitats is urgently needed.

## 1. Introduction

Wild birds represent a sentinel reservoir for a wide range of viral and bacterial pathogens, with the potential to infect domestic birds and disseminate over long distances in a short time [1]. Therefore, wild birds play a crucial role in the introduction, maintenance, and dissemination of pathogens globally, including zoonotics [2,3,4]. Several studies have indicated the transmission of antimicrobial resistant (AMR) bacteria from sewage, manure, contaminated water, and feeds from domestic birds as well as other animal and human sources to wild birds. These populations can then become reservoirs, recombination melting pots, and potential long-distance spreaders of antibiotic-resistant bacteria and resistance genes [5,6,7,8]. Furthermore, wild birds, especially waterfowl, represent the natural reservoir for influenza A viruses (IAV), which mostly present in the low pathogenic (LPAI) forms, including the H5 and H7 subtypes that were transmitted and maintained in domestic birds, to convert to high pathogenic avian influenza (HPAI) [9,10]. However, wild birds could also be a direct source of HPAI as in the case of the introduction of H5N1 and H5N8 to Egypt [11]. The other way around has been reported in outbreaks of HPAI-H5N1 in Asia, where HPAI subtypes have spilled over into wild bird populations from domestic birds in live bird markets (LBM) [12]. 

Multiple IAV subtypes (HPAI-H5N1, HPAI-H5N8, and LPAI-H9N2) are co-circulating in Egypt, resulting in huge economic losses. Notably, both HPAI-H5N1 and H5N8 were first detected in wild birds from a wetland in the Damietta Governorate in northern Egypt shortly before spreading to domestic birds [13,14,15]. In contrast, LPAI-H9N2 was first detected in domestic birds in Giza [16]. However, recent evidence of H9N2 reassortment with Eurasian AIVs circulating in wild birds was detected in pigeons, presumably due to co-infection with viruses from poultry and migratory waterfowl [17]. Wild migratory birds land in wetland areas in Egypt that are close to human activities including backyard and commercial farming, especially on the northern coast. These areas are where most of the cases have been confirmed [11].

The presence of antimicrobial resistance (AMR) in wild birds screened in Egypt has been confirmed with high homology, with samples collected from adjacent water, and both lack the homology with the human isolates [18]. Despite the relatively low number of samples, a recent study [18] suggests that water contaminated with domestic animal sewage could be a possible source of antimicrobial genes in bacteria found in wild birds.

The incidence rate of avian influenza in Egypt is particularly high in LBM as compared to backyard and commercial farms sampled birds. LBMs are very common and widely distributed all over Egypt due to the cultural preference for the consumption of freshly slaughtered poultry [15,19,20]. Some LBMs in Egypt sell migratory birds since trapping of such birds is common amongst local communities for commercial sale in LBM in the north coast cities. This is where they come into close contact with domestic birds [13].

Taken together, wild birds and LBM represent an important hub for avian influenza epidemiology and a potential reservoir for AMR in Egypt. In the present study, samples were collected from wild birds sold at LBM in the north coastal Governorates and tested for the presence of IAV and antimicrobial resistant *enterobacteriaceaeis*.

## 2. Results

### 2.1. Prevalence and Genetic Characterization of Influenza A viruses

Ten birds out of 148 were positive (6.76%) for the IAV M gene by real-time PCR. When subtyped, one remained untyped, four were H5N8, four were H9N2, and one was H6N2 (Supplementary Figure S1). No co-infection was detected in any of the collected samples. All positive samples were detected in Northern Shoveler birds (Spatula clypeata) sampled in Gamasa on December 2018 showing mild diarrhoea but with absence of respiratory or neurological clinical signs (Table 1) (Supplementary Table S1). 

**Figure 1:**
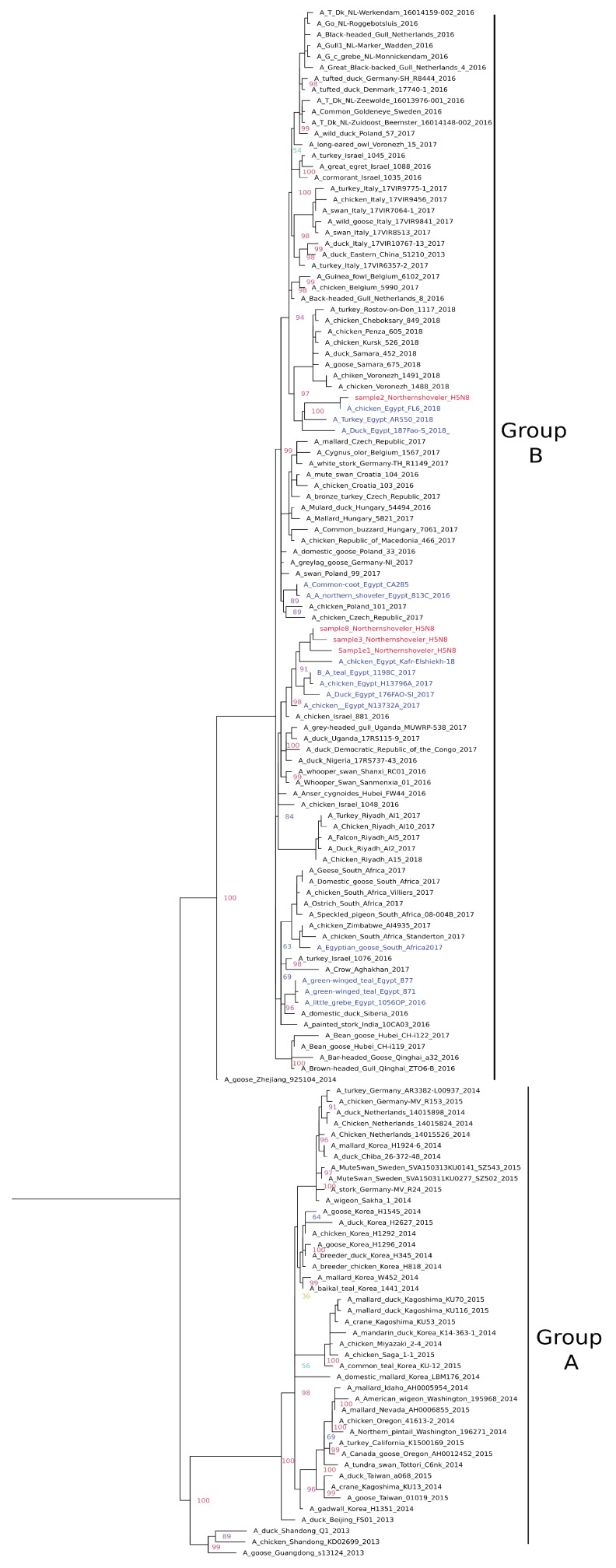
Phylogenetic relationships of HPAI-H5N8 (2.3.4.4) haemagglutinin (HA) gene of representative AIVs. The tree was generated by the maximum likelihood method and bootstrapped with 1000 replicates using the GTR substitution model with gamma distribution. Viruses characterized in this study are indicated in red and other Egyptian strains in blue.

**H5N8:** Phylogenetic analysis of the full HA gene of the four H5N8 viruses indicated that they clustered together within clade 2.3.4.4 group B (Figure 1) with recently isolated Egyptian H5N8 strains from domestic birds. Sample 2 clustered with 2018 strains with the characteristic amino acid substitution 175L previously reported in recent Egyptian samples [21]. All four samples possessed multibasic cleavage sites in the motif PLREKRRKR/GLF and six potential glycosylation sites at positions 10, 23, 165, 286, 483, and 542 (H5 numbering). All samples had Q222 and G224 that indicated preferential binding to α2-3sialic acidlinkages (avian receptors). The mutations R72S and L129S were detected in the receptor binding sites of samples 1 and 2 and samples 1, 3, and 8, respectively. Mutation T140A was detected in antigenic site A of sample 2. Further mutation at position Y252N was found in sample 2.

**Figure 2:**
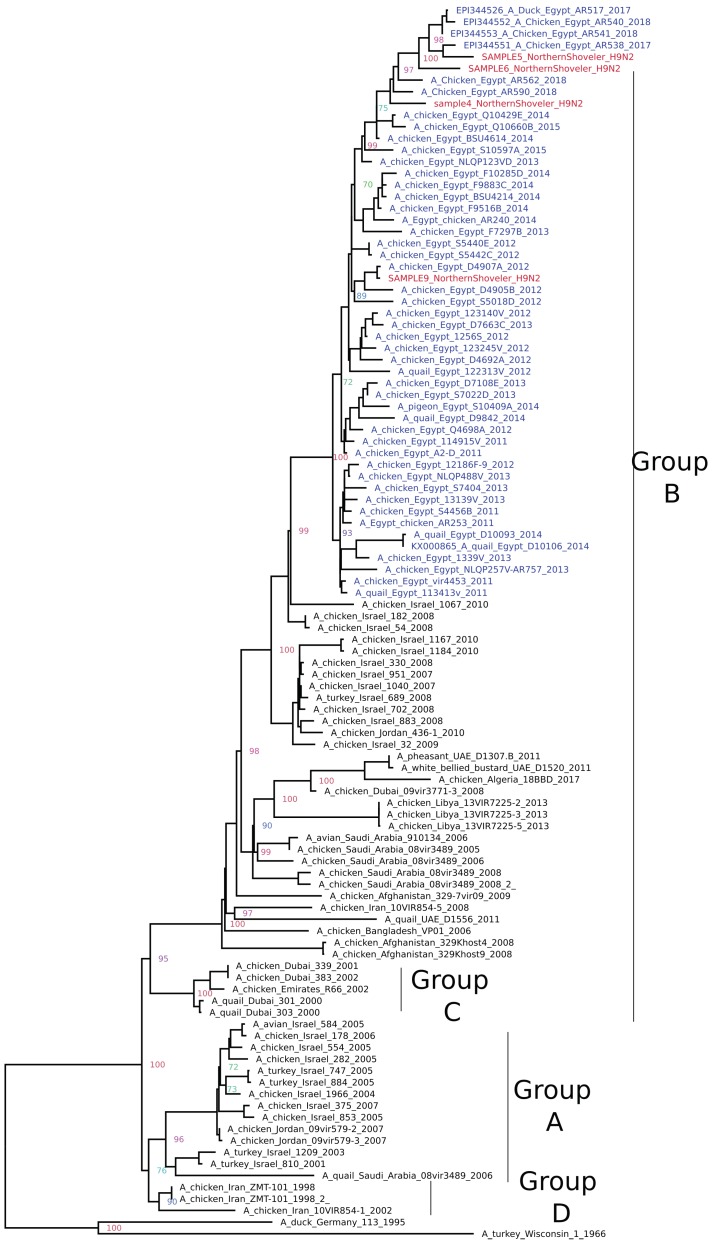
Phylogenetic relationships of H9 HA gene of representative AIVs. The tree was generated by the maximum likelihood method and bootstrapped with 1000 replicates using a GTR substitution model with gamma distribution. Viruses characterized in this study are indicated in red and other Egyptian strains in blue.

**H9N2:** Phylogenetic analysis of the full HA gene of the four H9N2 viruses revealed clustering within group B of G1-like Eurasian sub-lineages (A/quail/Hong Kong/G1/97-like). Sample 4,5, and 6 clustered with the most recent Egyptian strains available on GenBank, while sample 9 clustered with earlier strains circulated in 2012 (Figure 2). All four samples possessed monobasic cleavage sites in the motif PARSSR/GLF. Mutations H191 and L234 have been detected in samples 4, 6, and 9, which indicated a preference of2-6α sialic acid receptor (human-like). Alternatively, sample 5 possessed Q234, which is typical of an avian H9N2 signature. No other significant mutations were detected in comparison to other Egyptian H9N2 strains except mutation T198 in sample 6, which was located at the receptor-binding site. 

**Figure 3:**
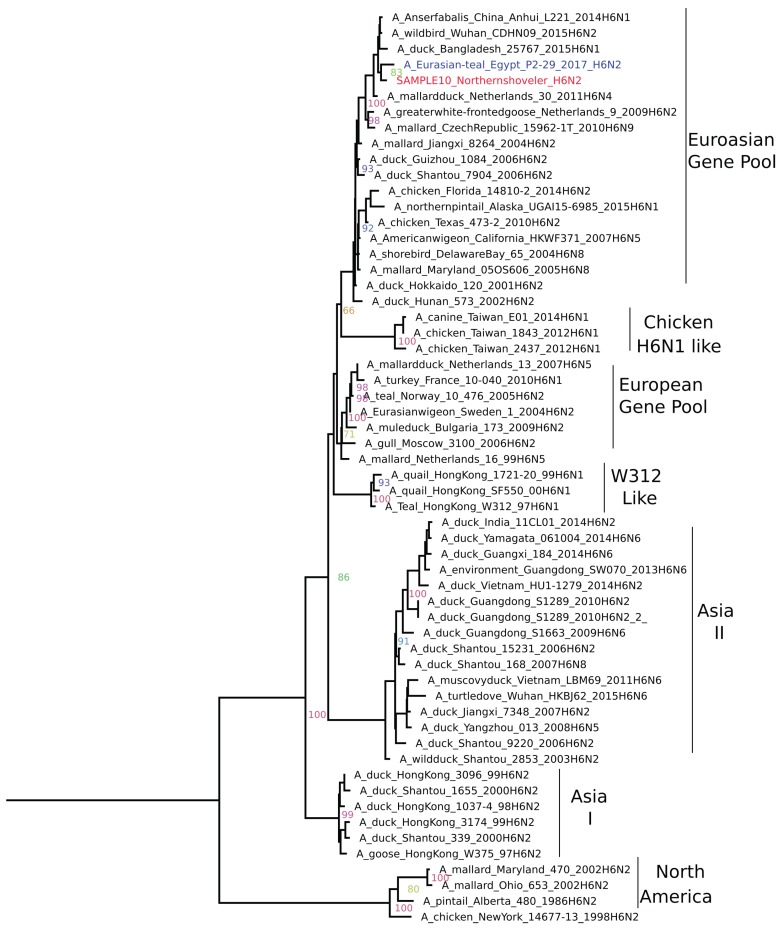
Phylogenetic Relationships of H6N2 HA gene of representative AIVs. The tree was generated by the maximum likelihood method and bootstrapped with 1000 replicates using a GTR substitution model with gamma distribution. Viruses characterized in this study are indicated in red and other Egyptian strains in blue.

**H6N2:** Phylogenetic analysis revealed grouping of the H6N2 positive sample with the Eurasian gene pool (Figure 3) represented by (A/duck/Hunan/573/2002(H6N2), as previously described [22]. BLAST analysis showed a close relationship (> 98% nucleotide homology) to a recent wild bird virus from China (A/wild bird/Wuhan/CDHN09/2015 (H6N2) and water wildfowl virus from Korea (A/water wildfowl/Korea/M129/2014(H6N1) and Egyptian H6N2 (A/Eurasian-teal/Egypt/P2-29/2017). This is suggestive of a Eurasian wild bird origin. Genome analysis revealed that the H6N2 viruses are low pathogenic to chickens with a monobasic cleavage site (PQIETR/GLF).The presence of Q226 and G228 (H3 numbering) on HA indicated a preference of avian (α2,3-linked sialic acid receptors). Using NetNGlyc 1.0 server six potential, N-linked glycosylation sites in HA were identified. 

### 2.2. Prevalence and Antibiotic Resistance of the Enterobacteriaceae

Out of 148 live wild bird samples, *E. coli* was recovered from 55 samples, while Salmonella was recovered from 30 samples. Nine of all samples showed Salmonella and *E. coli* co-infection (Supplementary Table S1). Unlike influenza, *E. coli* and Salmonella were isolated from almost all the wild bird species sampled (Table 1).

***E. coli*:** Serological identification revealed presence of O1, O78, O121, and O26 in 5, 11, 4, and 3 of *E. coli* positive samples, respectively (Table 2)**.** O1 and O78 serotypes are usually associated with avian pathogenic *E. coli* (APEC), while O121 and O26 are considered as potentially producing lethal toxins to humans. Enteropathogenic *E. coli* (EPEC), Enterotoxigenic *E. coli* (ETEC), and Enterohemorrhagic *E. coli* (EHEC) are widely prevalent in *E. coli* positive birds, while Enteroinvasive *E. coli* (EIEC) was only recovered from one bird (Supplementary Table S1). 

**Salmonella:***S. Enteriditis* and *S. Typhimurium* were recovered from 4 and 3 apparently healthy wild birds, respectively (Supplementary Table S1). 

**Other Enterobacteriaceae:***Klebsiella pneumoniae* has been recovered from 8 samples collected from apparently healthy birds. Enterobacter from 37 birds. Proteus from 44 birds and Citrobacter from 17 birds (Table 1) and (Supplementary Table S1).

**Antibiotic resistance:** Seven antibiotics that were considered as Highest Priority Critically Important Antimicrobials for human medicine (WHO-CIA) and commonly used in Egypt were selected and tested by all positive E. coli and Salmonella positive samples. Data showed that 81%, 74%, 81%, 65.5%, 84.5%, 94.8%, and 94.8% of *E. coli* isolates and 29%, 29%, 22.6%, 25.8% 41.9%, 38.7%, and 41.9% of Salmonella isolates were resistant to Ampicillin, Ciprofloxacin, Doxycycline, Erythromycin, Streptomycin, Tetracycline, and Amoxicillin, respectively (Table 3). 

## 3. Discussion

Wild birds represent the natural host of every known subtype of type A influenza, with the exception of H17N10 and H18N11, which were reported solely in bats [23]. Wild birds are responsible for the transcontinental spread of influenza viruses over thousands of kilometers and spill over to domestic birds and threaten human health. Few AIV subtypes become as well adapted to poultry as the panzootic goose/Guangdong lineage H5Nx viruses, the recent Chinese H7N9 viruses, and multiple Eurasian H9N2 lineages and present a major threat to veterinary and human health, while others quickly disappeared [24].

After long journeys from Europe and Asia, millions of exhausted wild birds rest in the wetlands of northern Egypt, especially around where the river Nile drains into the Mediterranean Sea. In this area, the trapping of wild birds is a very ancient and common practice for commercial sale in live bird markets [11]. Both residences of wild birds and offering them for sale in live bird markets represent a considerable risk for the spillover of the infection to domestic birds and possibly vice versa. 

In the present study, samples were collected from 148 trapped wild birds offered in LBM, representing 14 wild bird species. Only 10 Northern shoveler birds were positive for type A influenza virus as evidenced by real-time PCR. The Northern shoveler (Spatula clypeata) is considered an important AIV reservoir and is known to have high rates of AIV prevalence [25,26]. To our knowledge, 12 different AIV subtypes have been recovered from Northern shoveler in Egypt and these include H1N1, H2N8, H5N2, H6N2, H7N1, H7N3, H7N7, H7N9, H10N1, H10N4, H10N7, and H10N9 [20,26,27,28,29]. All of them present in low pathogenic forms. Alternatively, in the present study, HPAI-H5N8, LPAI-H9N2, and LPAI-H6N2 subtypes have been recovered from 4, 4, and 1birds, respectively. Hemagglutinin gene sequencing and phylogentic analysis showed higher similarity and clustering with currently circulating H5N8, H9N2, and H6N2 genotypes in domestic fowl. The same findings were previously reported in Egypt [26], where H5N1 and H9N2 viruses were detected in migratory mallards and were genetically closely related to AIV circulating in domestic poultry. In the same context, HPAI has been occasionally reported in wild birds near to affected poultry flocks, but these bird have played a minor or no role in the dissemination of the virus [30]. We believe that Northern shoveler acquire enzootic influenza infection via direct contact with domestic birds in LBM with the absence of clinical signs, which was previously reported [26]. Furthermore, we reported that all positive birds sampled on the same day from the same market had influenza with very high similarity to enzootic viruses, and this scenario suggests a single source of infection. In contrast, the pandemic H5N1 and H5N8 introduced to Egypt in late 2005 and 2016, respectively, were first detected in a Eurasian green-winged teal (Anas crecca) which was found dead in the same area [13,31]. In this case, viruses spread to domestic birds where they evolved and became markedly diverged from the original virus introduced via wild birds. Altogether, it is unlikely that captured infected wild birds can play a role in the dissemination of the virus to other destinations. However, it is worth mentioning that it is still a considerable risk factor of exporting enzootic H5N8 and H9N2 via migration. Further, wild birds in LBM could act as mixing vessels for different influenza subtypes that may lead to reassortment. Different surveillances strategies should be developed to find out whether the enzootic viruses are able to disseminate to wild birds from LBM or in their free habitat as well. Environmental sampling should include fresh fecal sampling and onsite birds sampling is required for insight analysis of the ability of domestic birds to disseminate enzootic viruses to wild birds in their habitat. 

Wild birds play a crucial role as potential reservoirs of enteric human and animal pathogens and vectors of antimicrobial resistance transfer. Information regarding the prevalence of bacterial pathogens and their antimicrobial pattern is limited for the wild birds in Egypt. In the present study, profiles of *Enterobacteriaceae* members were investigated in all sampled wild birds using standard methods. Fourteen members of the *Enterobacteriaceae* family were recovered. *E. coli* and Salmonella were recovered from 37.2% and 20.2% of all tested birds, respectively. Serotyping of the isolated *E. coli* revealed the wide prevalence of certain *E. coli* serotypes, which are more frequently associated with avian Collibacillosis such as O1 and O78 [32], and Shiga Toxin-Producing serotypes such as O121, O91, and O26 [33,34] were also recovered. Potentially, a higher prevalence of Salmonella spp. has been recovered in this study with a prevalence of 20.2% (n=30/148), when comparing to previous studies [35,36,37]. Furthermore, a high prevalence of both *E.coli* and Salmonella were detected in waterfowl, especially in the Northern Shoveler, where 38.2% and 32.4% of tested samples were positive for *E.coli* and Salmonella, respectively, with highly diversified *E. coli* and salmonella serovars, demonstrating the possible particular importance of the Northern shoveler in the dissemination of bacterial pathogens of human and veterinary importance as well as AIV in Egypt.

Although the wild birds were not directly exposed to antimicrobial agents, they get infected [7,38] and act as a reservoir and disseminator of resistant bacteria [39,40]. The WHO has categorized the antimicrobial reagents into critically important, highly important, and important antimicrobials [41]. In the present study, seven of the most commonly used critically important antimicrobials (CIA) in Egypt were selected to test all *E. coli* and Salmonella isolates. In the present study, resistance to CIA occurred in 66%–96% of *E. coli* strains and 23%–43% of Salmonella strains. The high rate of resistance of *E. coli* isolates to Streptomycin, tetracycline, and Amoxicillin (85%, 96%, and 96%, respectively) compared to 42%, 21%, and 26.3%, respectively, which was recently detected in wild birds in Switzerland, reflects the role of wild birds as a mirror for the unwise use of antimicrobial agents [7].

## 4. Materials and Methods 

### 4.1. Sample Collection

148 samples were collected from migratory birds offered for sale in LBM (114 from Damietta and 34 from Gamasa) during the winter season (between December 2018 and February 2019) (Table 1 and Supplementary Table S1). Both cities are located atthe north-eastern corner of the Nile delta. Damietta is located between the Damietta branch of the river Nile and Lake Manzala on the northern coast of Egypt. Gamasais located on the northeast coast toward the west of Damietta branch. This area hosts significant numbers of overwintering waterfowl each year andis frequented by poultry from surrounding households and commercial poultry farming. Furthermore, this area had positive influenza cases in domestic birds just before the sampling period, as confirmed by the Reference Laboratory for Veterinary Quality Control on Poultry Production (RLQP). The sample size was limited to the agreement of fishermen or bird sellers. However, to guide field researchers and ensure effective sampling, only live migratory birds that were caged or tied close to a live bird and offered for sale in LBM were sampled. Representative images of sampled birds and their clinical signs were reported. Two cloacal swabs were collected from each bird, the first of which was placed individually in cryovials containing viral transport medium for IAV detection. The second swab was kept in non-selective media for bacterial isolation and identification. Samples were stored on ice once collected and were transported rapidly to RLQP for laboratory processing. The sampling plan and experimental procedures were reviewed and approved by RLQP Scientific and Ethics committee (Agreement No. 2429), in accordance with guidelines of the Ministry of Agriculture and Land Reclamation and the Ministry of the Environment, Egypt.

### 4.2. Influenza Molecular Diagnosis

Swabs from each bird were individually subjected to RNA extraction using a QIAamp viral RNA mini kit (Qiagen, GmbH, Hilden, Germany) in accordance with the manufacturer’s instructions and extracted RNA quantity and purity were measured by Nano DropTM 2000 spectrophotometer. Reverse transcription and amplification were performed using the QuantiTect Probe RT-PCR kit (Qiagen, GmbH, Hilden, Germany) and analyzed using the Mx3005P real-time PCR system (Stratagene, La Jolla, CA, USA). The primers and probes that were used to amplify the AIV matrix gene (M) and the thermal profile were previously described [42]. Positive samples for the M gene were tested for HA- H5, H6, H7, and H9 in accordance with the procedures previously described by [43], [44], [45], and [46], respectively. The neuraminidase N1, N2, and N8 were tested as previously described by [47] and [48]. For molecular pathotyping and confirmation of PCR subtyping, a pan-HA RT-PCR targeting the HA0cleavage site of influenza A viruses followed by direct sequencing were used, as previously described by [49]. Neuraminidase subtyping was confirmed by pan-NA RT-PCR assay followed by direct sequencing, as previously described by [50]. Positive samples were subjected for Newcastle disease virus and coronavirus detection by quantitative RT-PCR, as previously described in [51] and [52], respectively.

### 4.3. Virus Isolation and Full HA Gene Sequencing

One hundred μl of the original cloacal swabs were inoculated once into embryonated chicken eggs and allantoic fluids were harvested in accordance with OIE recommendations [53].Collected allantoic fluids were tested for AIV by the haemagglutination (HA) assay and PCR targeting of the M gene before full HA sequencing. In brief, RNA was extracted using a QIAamp viral RNA mini kit (Qiagen, GmbH, Hilden, Germany) in accordance with the manufacturer’s guidelines. The first-strand cDNA was synthesized using SuperScript III reverse transcriptase (Invitrogen, Waltham, MA, USA) usingUni-12 universal primer [54]. Synthesised cDNA was amplified with specific primers that cover all HA genes [54]. PCR products of expected sizes were purified from agarose using a QIAquick Gel Extraction Kit (Qiagen, GmbH, Hilden, Germany) in accordance with the manufacturer’s instructions. Sequence reactions were performed using the Big Dye Terminator Version 3.1 cycle Sequencing Kit (Applied Biosystems, Foster City, CA, USA). Sequence reactions were purified using Centrisep spin columns (Thermo Fisher, Waltham, MA, USA) and were then analyzed using the 3130 Genetic Analyzer (Applied Biosystems, USA). The retrieved sense and antisense sequences were assembled and trimmed and a consensus was generated using the Geneious® software, version11.0.5 [55]. Full HA sequences of all samples were compared with the Pan HA sequence results, which were verified using an online Basic Local Alignment Search Tool (BLAST) (https://blast.ncbi.nlm.nih.gov/Blast.cgi).

To perform phylogenetic analysis, full HA sequences of H5, H6, and H9 sequences epresenting different genotypes were retrieved from GenBank and GSAID, with special reference to genotypes recently found circulating in Egypt and neighboring countries, and aligned with sequences from the present study. The alignment was performed with Clustal W in MEGA software version 7 [56]. Phylogenetic trees were constructed with the same software using maximum likelihood, with the substitution model general time-reversible with gamma-distribution (GTR+G) and 1000 bootstrap replicates. The trees were viewed using Fig tree V1.4.2 (http://tree.bio.ed.ac.uk/software/figtree/).

### 4.4. Bacteriological Isolation, Identification, and Antibiotic Resistance Profiling

Cloacal swab samples were placed in tubes containing buffered peptone water (Oxoid, Basingstoke, UK) and transported in an icebox to the laboratory in the RLQP-Gamasa branch. Bacteriological examinations for the detection of Enterobacteriaceae were performed in accordance with the reference procedure [57]. The biochemically confirmed *E. coli* and Salmonella isolates were serologically identified in accordance with [58] and [59], respectively. Unidentified isolates were subjected to further biochemical tests in accordance with [60]. For antimicrobial susceptibility testing, seven critically important antimicrobials [41] that are commonly used in Egypt were tested using the agar disc diffusion method on Muller Hinton agar plates in accordance with the reference procedure [61].

## 5. Conclusions

Altogether, wild birds in LBM represent a potential reservoir for influenza viruses and AMR, which represents a big risk, especially if we bear in mind that a lot of domestic birds that are offered for sale at LBM are mainly used for restocking, which will lead to further dissemination in the backyard system. However, it is still not fully confirmed whether wild birds acquired enzootic AIV and CIA resistance from other domestic birds in the LBM or through the intake of polluted water or food contaminated with human or animal waste in their habitat or from backyard birds near to their habitat. On that basis, further epidemiological studies include environmental samples from wild birds’ habitat, onsite wild birds sampling, plus samples from surrounding domestic birds in backyards, LBM, and from sellers using appropriate sanitation measures. This is necessary to understand the mode of transmission of resistant bacteria and enzootic AIV to wild birds and their ability to maintain and disseminate infection into the environment. Further, the high prevalence of antibiotic-resistant bacteria and presence of diversified *E. coli* serotypes, Salmonella pathotypes, and AIV in some species, such as Northern shoveler, which is considered a very common migratory bird that lands in Egypt for about four months per year, highlights the importance of providing a list of the number and species of the sampled birds in all future studies [11] and strengthens the importance of the directed surveillance in particular important wild birds. 

## Figures and Tables

**Table 1:** Sampled wild birds’ species with the prevalence of influenza viruses and *Enterobacteriaceae*.

Birds Common Name (Scientific Name)	No.	AIV (Subtypes)	Isolated Bacteria									
			E. coli	Salmonella	K. pneumoniae	Citrobacter		Enterobacter			Proteus	
						Freundii	Diversus	Aerogenes	Agglomerans	Cloacae	Mirabilis	Vulgaris
Northern Shoveler (Spatula clypeata)	34	10_ H6N2 (1), H5N8 (4), H9N2 (4) and one untyped	13 (38.2%)	11 (32.4%)	2 (5.9%)	2 (5.9%)	1 (2.9%)	5 (14.7%)	2 (5.9%)	-	9 (26.5%)	2 (5.9%)
Common pochard (Aythya ferina)	16	-	6 (37.5%)	2 (12.5%)	-	2 (12%)	1 (6.2%)	3 (18.75%)	2 (12.5%)	-	4 (25%)	2 (12.5%)
Northern pintail (Anas Acuta)	33	-	12 (36.4%)	3 (9.1%)	3 (9.1%)	3 (9.1%)	1 (3%)	5 (15.2%)	-	1 (3%)	6 (18.2%)	4 (12.1%)
Common teal(Anas crecca)	19	-	8 (42.1%)	-	-	2 (10%)	1 (5.2%)	3 (15.8%)	1 (5.25%)	-	4 (21.1%)	1 (5.25%)
Common moorhen (Gallinula chloropus)	18	-	6 (33.3%)	2 (11.1%)	1 (5.6%)	-	-	4 (11.1%)	2 (11.1%)	-	4 (11.1%)	-
Mallard ducks (Anas platyrhynchos)	3	-	1 (33.3%)	-	-	-	-	-	-	-	-	-
Purble Swamphens (Porphyrio porphyrio)	2	-	1 (50%)	-	1 (50%)	-	-	-	-	-	-	-
Garganey (Spatula querquedula)	7	-	3 (42.9%)	2 (28.57)	-	-	-	3 (42.9%)	2 (28.5%)	-	1 (14.3%)	-
Eurasian coot (Fulicaatra)	2	-	-	2 (100%)	-	-	-	-	-	1 (50%)	1 (50%)	2 (50%)
Glossy Ibis (Plegadis falcinellus)	1	-	1 (100%)	-	-	1 (100%)	-	-	-	-	-	-
Common redshank (Tringa tetanus)	7	-	2 (28.6%)	3 (42.9%)	-	3 (42.9%)	-	-	1 (14.3%)	-	2 (28.6%)	-
Common quail (Coturnixcoturnix)	3	-	1 (33.3%)	3 (100%)	1 (33.3%)	-	-	1 (33.3%)	-	-	-	-
Ruff (Philoma chusPugnax)	2	-	1 (50%)	1 (50%)	-	-	-	-	-	-	2 (100%)	-
Spur-winged Plover (Vanellu sspinosus)	1	-	-	1 (100%)	-	-	-	1 (100%)	-	-	-	-
Total	148	10	55	30	8	13	4	25	10	2	33	11

**Table 2:** Salmonella and *E. coli* serotypes prevalence in different wild birds’ species.

Sampled Bird	E. coli Serotype	Salmonella Serotype
Northern Shoveler	O1 (2), O78(3), O121(1), O128(2), O146, O158, O153, O2, O91	S. Kentucky (3), Molade, Enteritidis (2), Papuana, Typhimurium, Larochelle, Tsevie, Infantis
Common pochard	O78 (2), O55, O158, O125, O2	S. Tamale, Infantis
Northern pintail	O1, O78, O121, O91, O55, O113, O2(2), O128	S. Molade, Wingrove, Kentucky
common teal	O128, O78 (2), O44, O146, O1, O124, O26	-
Moorhen	O91, O26, O2, O121 (2), O78	S. untyped, Papuana7
Mallard Ducks	O26 (1)	-
Purble Swamphens	O1(1)	-
Garganey	O119, O146, O158	S. Typhimurium, Tamale
Coot	-	S. Papuana, Labadi
Glossy Ibis	O78(1)	-
Common redshank	O2, O125	S. Enteritidis, Kentucky, Tsevie
Common Quail	O128	3_S. Typhimurium (1), S. Enteritidis (2)
Ruff	O55	S. Inganda
Spur-winged Plover	-	S. Kentucky
surface swabs	O78, O91, O124	S. Kentucky

**Table 3:** Frequencies of *E. coli* and Salmonella isolates resistance to some of the Critically Important Antimicrobials commonly used in Egypt.

Antimicrobial Agent	E. coli Isolates (55)		Salmonella Isolates (30)	
	Sensitive NO. (%)	Resistant NO. (%)	Sensitive NO. (%)	Resistant NO. (%)
Ampicillin	10 (18%)	45 (82%)	21 (70%)	9 (30%)
Ciprofloxacin	14 (26%)	41 (75%)	21 (70%)	9 (30%)
Doxycycline	10 (18%)	45 (82%)	23 (77%)	7 (23%)
Erythromycin	19 (35%)	36 (66%)	22 (73%)	8 (27%)
Streptomycin	8 (15%)	47 (85%)	17 (57%)	13 (43%)
Tetracycline	2 (4%)	53 (96%)	18 (60%)	12 (40%)
Amoxicillin	2 (4%)	53 (96%)	17 (57%)	13 (43%)

## Supplementary Materials

The following are available online at https://www.mdpi.com/2076-0817/9/3/196/s1, Table S1: List of samples information.

## References

[1] Bonnedahl J., Järhult J.D. (2014). Antibiotic resistance in wild birds. Ups. J. Med. Sci..

[2] Hamer S.A., Lehrer E., Magle S.B. (2012). Wild Birds as Sentinels for Multiple Zoonotic Pathogens Along an Urban to Rural Gradient in Greater Chicago, Illinois. Zoonoses Public Health.

[3] Cooper J.E. (1990). Birds and zoonoses. Int. J. Avian. Sci..

[4] Benskin C.M., Wilson K., Jones K., Hartley I.R. (2009). Bacterial pathogens in wild birds: A review of the frequency and effects of infection. Biol. Rev..

[5] Nelson M., Jones S.H., Edwards C., Ellis J.C. (2008). Characterization of Escherichia coli populations from gulls, landfill trash, and wastewater using ribotyping. Dis. Aquat. Organ..

[6] Dolejska M., Literak I. (2019). Wildlife Is Overlooked in the Epidemiology of Medically Important Antibiotic-Resistant Bacteria. Antimicrob. Agents Chemother..

[7] Zurfluh K., Albini S., Mattmann P., Kindle P., Nüesch-Inderbinen M., Stephan R., Vogler B.R. (2019). Antimicrobial resistant and extended-spectrum beta-lactamase producing Escherichia coli in common wild bird species in Switzerland. Microbiology Open.

[8] Smith S., Wang J., Fanning S., McMahon B.J. (2014). Antimicrobial resistant bacteria in wild mammals and birds: A coincidence or cause for concern?. Ir. Vet. J..

[9] Banks J., Speidel E.S., Moore E., Plowright L., Piccirillo A., Capua I., Cordioli P., Fioretti A., Alexander D.J. (2001). Changes in the haemagglutinin and the neuraminidase genes prior to the emergence of highly pathogenic H7N1 avian influenza viruses in Italy. Arch. Virol..

[10] Steinhauer D.A. (1999). Role of hemagglutinin cleavage for the pathogenicity of influenza virus. Virology.

[11] Naguib M.M., Verhagen J.H., Samy A., Eriksson P., Fife M., Lundkvist Å., Ellström P., Järhult J.D. (2019). Avian influenza viruses at the wild-domestic bird interface in Egypt. Infect. Ecol. Epidemiol..

[12] Clark L., Hall J. (2006). Avian influenza in wild birds: Status as reservoirs, and risks to humans and agriculture. Ornithol. Monographs.

[13] Saad M.D., Ahmed L.S., Gamal-Eldein M.A., Fouda M.K., Khalil F., Yingst S.L., Parker M.A., Montevillel M.R. (2007). Possible avian influenza (H5N1) from migratory bird, Egypt. Emerg. Infect. Dis..

[14] Selim A.A., Erfan A.M., Hagag N., Zanaty A., Samir A.H., Samy M., Abdelhalim A., Arafa A.A., Soliman M.A., Shaheen M. (2017). Highly Pathogenic Avian Influenza Virus (H5N8) Clade 2.3.4.4 Infection in Migratory Birds, Egypt. Emerg. Infect. Dis..

[15] Abdelwhab E.M., Hassan M.K., Abdel-Moneim A.S., Naguib M.M., Mostafa A., Hussein I.T.M., Arafa A., Erfan A.M., Kilany W.H., Agour M.G. (2016). Introduction and enzootic of A/H5N1 in Egypt: Virus evolution, pathogenicity and vaccine efficacy ten years on. Infect. Genet. Evol..

[16] El-Zoghby E.F., Arafa A.S., Hassan M.K., Aly M.M., Selim A., Kilany W.H., Selim U., Nasef S., Aggor M.G., Abdelwhab E.M. (2012). Isolation of H9N2 avian influenza virus from bobwhite quail (Colinusvirginianus) in Egypt. Arch. Virol..

[17] Kandeil A., El-Shesheny R., Maatouq A., Moatasim Y., Cai Z., McKenzie P., Webby R., Kayali G., Ali M.A. (2017). Novel reassortant H9N2 viruses in pigeons and evidence for antigenic diversity of H9N2 viruses isolated from quails in Egypt. J. Gen. Virol..

[18] Ahmed Z.S., Elshafiee E.A., Khalefa H.S., Kadry M., Hamza D.A. (2019). Evidence of colistin resistance genes (mcr-1 and mcr-2) in wild birds and its public health implication in Egypt. Antimicrob. Resist. Infect. Control.

[19] Abdelwhab E.M., Selim A.A., Arafa A., Galal S., Kilany W.H., Hassan M.K., Aly M.M., Hafez M.H. (2010). Circulation of avian influenza H5N1 in live bird markets in Egypt. Avian Dis..

[20] El-Zoghby E.F., Aly M.M., Nasef S.A., Hassan M.K., Arafa A.S., Selim A.A., Kholousy S.G., Kilany W.H., Safwat M., Abdelwhab E.M. (2013). Surveillance on A/H5N1 virus in domestic poultry and wild birds in Egypt. Virol. J..

[21] Hassan K.E., El-Kady M.F., El-Sawah A.A.A., Luttermann C., Parvin R., Shany S., Beer M., Harder T. (2019). Respiratory disease due to mixed viral infections in poultry flocks in Egypt between 2017 and 2018: Upsurge of highly pathogenic avian influenza virus subtype H5N8 since 2018. Transbound. Emerg. Dis..

[22] Huang K., Bahl J., Fan X.H., Vijaykrishna D., Cheung C.L., Webby R.J., Webster R.G., Chen H., Smith G.J., Peiris J.S. (2010). Establishment of an H6N2 influenza virus lineage in domestic ducks in southern China. J. Virol..

[23] Wu Y., Wu Y., Tefsen B., Shi Y., Gao G.F. (2014). Bat-derived influenza-like viruses H17N10 and H18N11. Trends Microbiol..

[24] Peacock T.H.P., James J., Sealy J.E., Iqbal M. (2019). A Global Perspective on H9N2 Avian Influenza Virus. Viruses.

[25] Hill N.J., Takekawa J.Y., Cardona C.J., Ackerman J.T., Schultz A.K., Spragens K.A., Boyce W.M. (2010). Waterfowl Ecology and Avian Influenza in California: Do Host Traits Inform Us About Viral Occurrence?. Avian Dis..

[26] Kayed A.S., Kandeil A., Gomaa M.R., El-Shesheny R., Mahmoud S., Hegazi N., Fayez M., Sheta B., McKenzie P.P., Webby R.J. (2019). Surveillance for avian influenza viruses in wild birds at live bird markets, Egypt, 2014–2016. Influenza Other Respir. Viruses.

[27] Verhagen J.H., Lexmond P., Vuong O., Schutten M., Guldemeester J., Osterhaus A.D., Elbers A.R., Slaterus R., Hornman M., Koch G. (2017). Discordant detection of avian influenza virus subtypes in time and space between poultry and wild birds; Towards improvement of surveillance programs. PLoS ONE.

[28] Zhang Y., Aevermann B.D., Anderson T.K., Burke D.F., Dauphin G., Gu Z., He S., Kumar S., Larsen C.N., Lee A.J. (2017). Influenza Research Database: An integrated bioinformatics resource for influenza virus research. Nucleic Acids Res..

[29] Soliman A., Saad M., Elassal E., Amir E., Plathonoff C., Bahgat V., El-Badry M., Ahmed L.S., Fouda M., Gamaleldin M. (2012). Surveillance of avian influenza viruses in migratory birds in Egypt, 2003–2009. J. Wildl. Dis..

[30] Swayne D.E.H., Influenza D.A., Saif Y.M.B., Glisson J.R., Fadly A.M., McDougald L.R., Swayne D.E. (2003). Diseases of Poultry.

[31] Kandeil A., Kayed A., Moatasim Y., Webby R.J., McKenzie P.P., Kayali G., Ali M.A. (2017). Genetic characterization of highly pathogenic avian influenza A H5N8 viruses isolated from wild birds in Egypt. J. General Virol..

[32] Collingwood C., Kemmett K., Williams N., Wigley P. (2014). Is the Concept of Avian Pathogenic Escherichia coli as a Single Pathotype Fundamentally Flawed?. Front. Veterinary Sci..

[33] Crowe S.J., Bottichio L., Shade L.N., Whitney B.M., Corral N., Melius B., Arends K.D., Donovan D., Stone J., Allen K. (2017). Shiga Toxin-Producing E. coli Infections Associated with Flour. New Engl. J. Med..

[34] Feng P.C.H., Delannoy S., Lacher D.W., Bosilevac J.M., Fach P., Beutin L. (2017). Shiga Toxin-Producing Serogroup O91 Escherichia coli Strains Isolated from Food and Environmental Samples. Appl. Environ. Microbiol..

[35] Giacopello C., Foti M., Mascetti A., Grosso F., Ricciardi D., Fisichella V., Lo Piccolo F. (2016). Antimicrobial resistance patterns of Enterobacteriaceae in European wild bird species admitted in a wildlife rescue centre. Vet. Ital..

[36] Abulreesh H.H., Goulder R., Scott G.W. (2007). Wild birds and human pathogens in the context of ringing and migration. Ring. Migr..

[37] Magda A., Mohamad N., Maysa A., Merwad A., Rasha M., Rasha M., Rehab E. (2013). Prevalence of Enterobacteriaceae in wild birds and humans at Sharkia Province; With special reference to the genetic relationship between E. coli and Salmonella isolates determined by protein profile analysis. J. Am. Sci..

[38] Guenther S., Ewers C., Wieler L.H. (2011). Extended-Spectrum Beta-Lactamases Producing E. coli in Wildlife, yet Another Form of Environmental Pollution?. Front. Microbiol..

[39] Arnold K.E., Williams N.J., Bennett M. (2016). ‘Disperse abroad in the land’: The role of wildlife in the dissemination of antimicrobial resistance. Biol. Lett..

[40] Agnew A., Wang J., Fanning S., Bearhop S., McMahon B.J. (2016). Insights into antimicrobial resistance among long distance migratory East Canadian High Arctic light-bellied Brent geese (Branta bernicla hrota). Ir. Vet. J..

[41] WHO (2019). WHO List of Critically I Antimicrobials for Human Medicine (WHO CIA List).

[42] Spackman E., Senne D.A., Myers T.J., Bulaga L.L., Garber L.P., Perdue M.L., Lohman K., Daum L.T., Suarez D.L. (2002). Development of a real-time reverse transcriptase PCR assay for type A influenza virus and the avian H5 and H7 hemagglutinin subtypes. J. Clin. Microbiol..

[43] Londt B.Z., Nunez A., Banks J., Nili H., Johnson L.K., Alexander D.J. (2008). Pathogenesis of highly pathogenic avian influenza A/turkey/Turkey/1/2005 H5N1 in Pekin ducks (Anas platyrhynchos) infected experimentally. Avian Pathol..

[44] Lee M.S., Chang P.C., Shien J.H., Cheng M.C., Shieh H.K. (2001). Identification and subtyping of avian influenza viruses by reverse transcription-PCR. J. Virol. Methods.

[45] Slomka M.J., Pavlidis T., Coward V.J., Voermans J., Koch G., Hanna A., Banks J., Brown I.H. (2009). Validated Real Time reverse transcriptase PCR methods for the diagnosis and pathotyping of Eurasian H7 avian influenza viruses. Influenza Other Respir. Viruses.

[46] Ben Shabat M., Meir R., Haddas R., Lapin E., Shkoda I., Raibstein I., Perk S., Davidson I. (2010). Development of a real-time TaqMan RT-PCR assay for the detection of H9N2 avian influenza viruses. J. Virol. Methods.

[47] Li L.H., Yu Z., Chen W.S., Liu S.L., Lu Y., Zhang Y.J., Chen E.F., Lin J.F. (2013). Evidence for H5 avian influenza infection in Zhejiang province, China, 2010–2012: A cross-sectional study. J. Thorac. Dis..

[48] Hoffmann B., Hoffmann D., Henritzi D., Beer M., Harder T.C. (2016). Riems influenza a typing array (RITA): An RT-qPCR-based low density array for subtyping avian and mammalian influenza a viruses. Sci. Rep..

[49] Gall A., Hoffmann B., Harder T., Grund C., Beer M. (2008). Universal primer set for amplification and sequencing of HA0 cleavage sites of all influenza A viruses. J. Clin. Microbiol..

[50] Gall A., Hoffmann B., Harder T., Grund C., Ehricht R., Beer M. (2009). Rapid and Highly Sensitive Neuraminidase Subtyping of Avian Influenza Viruses by Use of a Diagnostic DNA Microarray. J. Clin. Microbiol..

[51] Wise M.G., Suarez D.L., Seal B.S., Pedersen J.C., Senne D.A., King D.J., Kapczynski D.R., Spackman E. (2004). Development of a real-time reverse-transcription PCR for detection of newcastle disease virus RNA in clinical samples. J. Clin. Microbiol..

[52] Callison S.A., Hilt D.A., Boynton T.O., Sample B.F., Robison R., Swayne D.E., Jackwood M.W. (2006). Development and evaluation of a real-time Taqman RT-PCR assay for the detection of infectious bronchitis virus from infected chickens. J. Virol. Methods.

[53] OIE Chapter 3.3.4. Avian Influenza.2015. https://www.oie.int/fileadmin/Home/eng/Health_standards/tahm/3.03.04_AI.pdf.

[54] Hoffmann E., Stech J., Guan Y., Webster R.G., Perez D.R. (2001). Universal primer set for the full-length amplification of all influenza A viruses. Arch. Virol..

[55] Kearse M., Moir R., Wilson A., Stones-Havas S., Cheung M., Sturrock S., Buxton S., Cooper A., Markowitz S., Duran C. (2012). Geneious Basic: An integrated and extendable desktop software platform for the organization and analysis of sequence data. Bioinformatics.

[56] Tamura K., Stecher G., Peterson D., Filipski A., Kumar S. (2013). MEGA6: Molecular evolutionary genetics analysis version 6.0. Mol. Biol. Evol..

[57] Ewing W.H. (1986). Edwards and Ewing’s Identification of Enterobacteriaceae.

[58] Kok T., Worswich D., Gowans E., Collee J., Fraser A., Marmion B., Simmons A. (1996). Some serological techniques for microbial and viral infections. Practical Medical Microbiology.

[59] Grimont P.A., Weill F.-X. (2007). Antigenic formulae of the Salmonella serovars. WHO Collaborating Centre Ref. Res. Salmon..

[60] Quinn P., Carter M., Markey B., Donnoly W., Leonard F. (2002). Veterinary Microbiology and Microbial Diseases 166-1117 Osney Mead.

[61] Finegold S., Martin S. (1982). Diagnostic Microbiology 6th ed the CV Mosby Company.

