# Supplementary material for: Wild Birds in Live Birds Markets: Potential Reservoirs of Enzootic Avian Influenza Viruses and Antimicrobial Resistant Enterobacteriaceae in Northern Egypt
Source: Pathogens. 2020 Mar 6;9(3):196. doi: 10.3390/pathogens9030196 (PMC7157678; doi:10.3390/pathogens9030196)

Supplementary Table 1: List of samples information.

Serial	Date of sampling	Cities	Birds Common name (Scientific name)	health status	AIV	Salmonella	E.coli	enterobacteriaceae
1	30/12/018	Gamasa	Northern Northern shoveler (Spatula clypeata)	Diarrhea	H5N8	Kentucky	-	Proteus mirabilis
2	30/12/018	Gamasa	Northern Northern shoveler (Spatula clypeata)	Diarrhea	H5N8	-	-	Proteus vulgaris
3	30/12/018	Gamasa	Northern Northern shoveler (Spatula clypeata)	Diarrhea	H5N8	Molade	O91 : H21	-
4	30/12/018	Gamasa	Northern Northern shoveler (Spatula clypeata)	Diarrhea	H9N2	-	-	Proteus mirabilis
5	30/12/018	Gamasa	Northern Northern shoveler (Spatula clypeata)	Diarrhea	H9N2	-	O128 : H2	Providencia rettgeri
6	30/12/018	Gamasa	Northern Northern shoveler (Spatula clypeata)	Diarrhea	H9N2	-	O1 : H7	Enterobacter aerogenes
7	30/12/018	Gamasa	Northern Northern shoveler (Spatula clypeata)	Diarrhea	-	-	-	Enterobacter agglomerans
8	30/12/018	Gamasa	Northern Northern shoveler (Spatula clypeata)	Diarrhea	H5N8	-	-	Proteus vulgaris
9	30/12/018	Gamasa	Northern Northern shoveler (Spatula clypeata)	Diarrhea	H9N2	-	O78	Serratia liquefaciens
10	30/12/018	Gamasa	Northern Northern shoveler (Spatula clypeata)	Diarrhea	H6N2	-	-	Enterobacter aerogenes
11	30/12/018	Gamasa	Northern Northern shoveler (Spatula clypeata)	Diarrhea	-	-	-	Enterobacter aerogenes
12	30/12/018	Gamasa	Northern Northern shoveler (Spatula clypeata)	Diarrhea	-	-	-	Proteus mirabilis
13	31/12/018	Damietta	Northern Northern shoveler (Spatula clypeata)	Diarrhea	-	-	-	Hafnia species
14	31/12/018	Damietta	Northern Northern shoveler (Spatula clypeata)	Diarrhea	-	-	O146 : H21	Providencia rettgeri
15	31/12/018	Damietta	Northern Northern shoveler (Spatula clypeata)	Diarrhea	-	-	-	Citrobacter freundii
16	31/12/018	Damietta	Northern Northern shoveler (Spatula clypeata)	Diarrhea	-	-	O1 : H7	Proteus mirabilis
17	31/12/018	Damietta	Northern Northern shoveler (Spatula clypeata)	Diarrhea	-	-	O158	Proteus mirabilis

18	31/12/018	Damietta	Northern Northern shoveler (Spatula clypeata)	Diarrhea	-	-	O153 : H2	-
19	31/12/018	Damietta	Northern Northern shoveler (Spatula clypeata)	Diarrhea	-	-	-	Enterobacter agglomerans
20	31/12/018	Damietta	Northern Northern shoveler (Spatula clypeata)	Diarrhea		-	-	Proteus mirabilis
21	31/12/018	Damietta	Northern Northern shoveler (Spatula clypeata)	Diarrhea	-	-	O2: H6	Enterobacter aerogenes
22	31/12/018	Damietta	Northern Northern shoveler (Spatula clypeata)	Diarrhea	-	-	-	Proteus mirabilis
23	31/12/018	Damietta	Northern Northern shoveler (Spatula clypeata)	Diarrhea	-	-	O128 : H2	Serratia liquefaciens
24	3/1/019	Damietta	Northern Northern shoveler (Spatula clypeata)	healthy	-	-	-	Klebsiella pneumoniae
25	3/1/019	Damietta	Northern Northern shoveler (Spatula clypeata)	healthy	-	Enteritidis	-	Proteus mirabilis
26	3/1/019	Damietta	Northern Northern shoveler (Spatula clypeata)	healthy	-	Papuana	-	Klebsiella pneumoniae
27	3/1/019	Damietta	Northern Northern shoveler (Spatula clypeata)	healthy	-	-	O121 : H7	Citrobacter diversus
28	3/2/019	Damietta	Northern Northern shoveler (Spatula clypeata)	healthy	-	Kentucky	-	Enterobacter aerogenes
29	3/2/019	Damietta	Northern Northern shoveler (Spatula clypeata)	healthy	-	Kentucky	O78	Proteus mirabilis
30	3/2/019	Damietta	Northern Northern shoveler (Spatula clypeata)	healthy	-	Typhimurium	O78	-
31	3/2/019	Damietta	Northern Northern shoveler (Spatula clypeata)	healthy	-	Larochelle	-	Providencia rettgeri
32	3/2/019	Damietta	Northern Northern shoveler (Spatula clypeata)	healthy	-	Enteritidis	-	Citrobacter freundii
33	9/12/018	Damietta	Common pochard (Aythyaferina)	Diarrhea	-	-	-	Enterobacter aerogenes
34	9/12/018	Damietta	Common pochard (Aythyaferina)	Diarrhea	-	Tamale	O55 : H7	Proteus mirabilis
35	30/12/018	Gamasa	Common pochard (Aythyaferina)	Diarrhea	-	Infantis	-	Enterobacter agglomerans
36	30/12/018	Gamasa	Common pochard (Aythyaferina)	Diarrhea	-	-	-	Mixed Culture
37	30/12/018	Gamasa	Common pochard (Aythyaferina)	Diarrhea	-	-	-	Citrobacter freundii
38	30/12/018	Gamasa	Common pochard (Aythyaferina)	Diarrhea	-	-	-	Proteus mirabilis

39	30/12/018	Gamasa	Common pochard (Aythyaferina)	Diarrhea	-	-	O78	Proteus vulgaris
40	30/12/018	Gamasa	Common pochard (Aythyaferina)	Diarrhea	-	-	-	Enterobacter aerogenes
41	30/12/018	Gamasa	Common pochard (Aythyaferina)	Diarrhea	-	-	O78	Proteus vulgaris
42	30/12/018	Gamasa	Common pochard (Aythyaferina)	Diarrhea	-	-	O158	Enterobacter aerogenes
43	30/12/018	Gamasa	Common pochard (Aythyaferina)	Diarrhea	-	-	O125 : H21	Proteus mirabilis
44	20/1/019	Damietta	Common pochard (Aythyaferina)	healthy	-	-	-	Enterobacter agglomerans
45	20/1/019	Damietta	Common pochard (Aythyaferina)	healthy	-	-	-	Providencia rettgeri
46	20/1/019	Damietta	Common pochard (Aythyaferina)	healthy	-	-	-	Citrobacter freundii
47	20/1/019	Damietta	Common pochard (Aythyaferina)	healthy	-	-	-	Citrobacter diversus
48	20/1/019	Damietta	Common pochard (Aythyaferina)	healthy	-	-	O2: H6	Proteus mirabilis
49	9/12/018	Gamasa	Northern pintail (Anas Acuta)	Diarrhea	-	-	-	Providencia rettgeri
50	9/12/018	Gamasa	Northern pintail (Anas Acuta)	Diarrhea	-	-	-	Hafnia species
51	23/12/018	Damietta	Northern pintail (Anas Acuta)	healthy	-	-	-	Klebsiella pneumoniae
52	25/12/018	Damietta	Northern pintail (Anas Acuta)	healthy	-	-	O121 : H7	Enterobacter aerogenes
53	25/12/018	Damietta	Northern pintail (Anas Acuta)	healthy	-	-	-	Providencia rettgeri
54	25/12/018	Damietta	Northern pintail (Anas Acuta)	healthy	-	-	O91 : H21	Proteus mirabilis
55	25/12/018	Damietta	Northern pintail (Anas Acuta)	healthy	-	-	-	Citrobacter freundii
56	30/12/018	Gamasa	Northern pintail (Anas Acuta)	Diarrhea	-	-	-	Enterobacter aerogenes
57	30/12/018	Gamasa	Northern pintail (Anas Acuta)	Diarrhea	-	-	O55 : H7	Proteus vulgaris
58	30/12/018	Gamasa	Northern pintail (Anas Acuta)	Diarrhea	-	-	O113 : H4	Mixed Culture
59	30/12/018	Gamasa	Northern pintail (Anas Acuta)	Diarrhea	-	-	-	Proteus mirabilis
60	30/12/018	Gamasa	Northern pintail (Anas Acuta)	Diarrhea	-	-	O78	Proteus mirabilis
61	30/12/018	Gamasa	Northern pintail (Anas Acuta)	Diarrhea	-	-	-	Enterobacter aerogenes
62	30/12/018	Gamasa	Northern pintail (Anas Acuta)	Diarrhea	-	-	-	Proteus mirabilis
63	30/12/018	Gamasa	Northern pintail (Anas Acuta)	Diarrhea	-	-	O2: H6	Serratia liquefaciens
64	30/12/018	Gamasa	Northern pintail (Anas Acuta)	Diarrhea	-	-	-	Hafnia species
65	31/12/018	Damietta	Northern pintail (Anas Acuta)	healthy	-	-	-	Citrobacter freundii
66	31/12/018	Damietta	Northern pintail (Anas Acuta)	healthy	-	-	-	Proteus mirabilis

67	31/12/018	Damietta	Northern pintail (Anas Acuta)	healthy	-	Molade	O1 : H7	Mixed Culture
68	31/12/018	Damietta	Northern pintail (Anas Acuta)	healthy	-	Wingrove	-	Enterobacter aerogenes
69	31/12/018	Damietta	Northern pintail (Anas Acuta)	healthy	-	Kentucky	O158	Klebsiella pneumoniae
70	31/12/018	Damietta	Northern pintail (Anas Acuta)	healthy	-	-	O91 : H21	Proteus mirabilis
71	31/12/018	Damietta	Northern pintail (Anas Acuta)	healthy	-	-	-	Mixed Culture
72	31/12/018	Damietta	Northern pintail (Anas Acuta)	healthy	-	-	-	Citrobacter diversus
73	3/1/019	Damietta	Northern pintail (Anas Acuta)	healthy	-	-	-	Enterobacter cloacae
74	3/1/019	Damietta	Northern pintail (Anas Acuta)	healthy	-	-	-	Proteus vulgaris
75	3/1/019	Damietta	Northern pintail (Anas Acuta)	healthy	-	-	O2: H6	Proteus vulgaris
76	3/1/019	Damietta	Northern pintail (Anas Acuta)	healthy	-	-	-	Klebsiella pneumoniae
77	3/1/019	Damietta	Northern pintail (Anas Acuta)	healthy	-	-	O128 : H2	Serratia liquefaciens
78	3/1/019	Damietta	Northern pintail (Anas Acuta)	healthy	-	-	-	Providencia rettgeri
79	20/1/019	Damietta	Northern pintail (Anas Acuta)	healthy	-	-	-	Enterobacter aerogenes
80	20/1/019	Damietta	Northern pintail (Anas Acuta)	healthy	-	-	-	Citrobacter freundii
81	20/1/019	Damietta	Northern pintail (Anas Acuta)	healthy	-	-	O2: H6	Proteus vulgaris
82	9/12/018	Gamasa	common teal (Anas crecca)	Diarrhea	-	-	-	Proteus mirabilis
83	9/12/018	Gamasa	common teal (Anas crecca)	Diarrhea	-	-	O44 : H18	Enterobacter aerogenes
84	31/12/018	Damietta	common teal (Anas crecca)	healthy	-	-	O78	Proteus mirabilis
85	31/12/018	Damietta	common teal (Anas crecca)	healthy	-	-	O128 : H2	Serratia liquefaciens
86	31/12/018	Damietta	common teal (Anas crecca)	healthy	-	-	-	Klebsiella ozaenae
87	31/12/018	Damietta	common teal (Anas crecca)	healthy	-	-	-	Hafnia species
88	31/12/018	Damietta	common teal (Anas crecca)	healthy	-	-	-	Mixed Culture
89	31/12/018	Damietta	common teal (Anas crecca)	healthy	-	-	O78	Proteus vulgaris
90	31/12/018	Damietta	common teal (Anas crecca)	healthy	-	-	O26 : H11	Enterobacter aerogenes
91	31/12/018	Damietta	common teal (Anas crecca)	healthy	-	-	-	Proteus mirabilis
92	31/12/018	Damietta	common teal (Anas crecca)	healthy	-	-	O124	Citrobacter diversus
93	31/12/018	Damietta	common teal (Anas crecca)	healthy	-	-	-	Enterobacter agglomerans
94	31/12/018	Damietta	common teal (Anas crecca)	healthy	-	-	-	Citrobacter freundii
95	31/12/018	Damietta	common teal (Anas crecca)	healthy	-	-	O1 : H7	Providencia rettgeri

96	20/1/019	Damietta	common teal (Anas crecca)	healthy	-	-	-	Citrobacter freundii
97	20/1/019	Damietta	common teal (Anas crecca)	healthy	-	-	O146 : H21	Enterobacter aerogenes
98	20/1/019	Damietta	common teal (Anas crecca)	healthy	-	-	-	Mixed Culture
99	20/1/019	Damietta	common teal (Anas crecca)	healthy	-	-	-	Proteus mirabilis
100	20/1/019	Damietta	common teal (Anas crecca)	healthy	-	-	-	Providencia rettgeri
101	25/12/018	Damietta	moorhen (Gallinulachloropus)	healthy	-	-	-	Enterobacter agglomerans
102	25/12/018	Damietta	moorhen (Gallinulachloropus)	healthy	-	-	O121 : H7	Proteus mirabilis
103	31/12/018	Damietta	moorhen (Gallinulachloropus)	healthy	-	untyped	O2: H6	Enterobacter aerogenes
104	31/12/018	Damietta	moorhen (Gallinulachloropus)	healthy	-	-	-	Mixed Culture
105	31/12/018	Damietta	moorhen (Gallinulachloropus)	healthy	-	Papuana	-	Proteus mirabilis
106	31/12/018	Damietta	moorhen (Gallinulachloropus)	healthy	-	-	O26 : H11	Serratia liquefaciens
107	31/12/018	Damietta	moorhen (Gallinulachloropus)	healthy	-	-	-	Enterobacter aerogenes
108	31/12/018	Damietta	moorhen (Gallinulachloropus)	healthy	-	-	-	Hafnia species
109	31/12/018	Damietta	moorhen (Gallinulachloropus)	healthy	-	-	-	Enterobacter aerogenes
110	31/12/018	Damietta	moorhen (Gallinulachloropus)	healthy	-	-	-	Serratia liquefaciens
111	31/12/018	Damietta	moorhen (Gallinulachloropus)	healthy	-	-	O91 : H21	Citrobacter freundii
112	31/12/018	Damietta	moorhen (Gallinulachloropus)	healthy	-	-	O78	Proteus mirabilis
113	31/12/018	Damietta	moorhen (Gallinulachloropus)	healthy	-	-	-	Enterobacter agglomerans
114	31/12/018	Damietta	moorhen (Gallinulachloropus)	healthy	-	-	-	Providencia rettgeri
115	31/12/018	Damietta	moorhen (Gallinulachloropus)	healthy	-	-	-	Klebsiella pneumoniae
116	31/12/018	Damietta	moorhen (Gallinulachloropus)	healthy	-	-	-	Proteus mirabilis
117	31/12/018	Damietta	moorhen (Gallinulachloropus)	healthy	-	-	O121 : H7	Citrobacter freundii
118	31/12/018	Damietta	moorhen (Gallinulachloropus)	healthy	-	-	-	Enterobacter aerogenes
119	31/12/018	Damietta	mallard ducks(Anas Platyrhynchos)	Diarrhea	-	-	-	Providencia rettgeri
120	31/12/018	Damietta	mallard ducks(Anas Platyrhynchos)	Diarrhea	-	-	O26 : H11	Proteus vulgaris
121	31/12/018	Damietta	mallard ducks(Anas Platyrhynchos)	Diarrhea	-	-	-	Serratia liquefaciens

122	31/12/018	Damietta	PurbleSwamphens (Porphyrioporphyrio)	healthy	-	-	O1 : H7	Mixed Culture
123	31/12/018	Damietta	PurbleSwamphens (Porphyrioporphyrio)	healthy	-	-	-	Klebsiella pneumoniae
124	9/12/018	Damietta	Garganey (Spatula querquedula)	healthy	-	-	-	Enterobacter agglomerans
125	9/12/018	Damietta	Garganey (Spatula querquedula)	healthy	-	-	O146 : H21	Hafnia species
126	3/2/019	Damietta	Garganey (Spatula querquedula)	healthy	-	-	O158	Proteus mirabilis
127	3/2/019	Damietta	Garganey (Spatula querquedula)	healthy	-	-	-	Enterobacter aerogenes
128	24/2/019	Damietta	Garganey (Spatula querquedula)	healthy	-	Tamale	-	Enterobacter aerogenes
129	24/2/019	Damietta	Garganey (Spatula querquedula)	healthy	-	Typhimurium	-	Enterobacter agglomerans
130	24/2/019	Damietta	Garganey (Spatula querquedula)	healthy	-	-	O119 : H6	Enterobacter aerogenes
131	9/12/018	Damietta	coot (Fulicaatra)	healthy	-	Papuana	-	Proteus mirabilis
132	9/12/018	Damietta	coot (Fulicaatra)	healthy	-	Labadi	-	Enterobacter cloacae
133	3/2/019	Damietta	Glossy Ibis (Plegadisfalcinellus)	healthy	-	-	O78	Citrobacter freundii
134	3/2/019	Damietta	Common redshank (Tringatotanus)	healthy	-	-	-	Citrobacter freundii
135	3/2/019	Damietta	Common redshank (Tringatotanus)	healthy	-	-	O2: H6	Proteus mirabilis
136	24/2/019	Damietta	Common redshank (Tringatotanus)	healthy	-	Enteritidis	-	Citrobacter freundii
137	24/2/019	Damietta	Common redshank (Tringatotanus)	healthy	-	Kentucky	-	Enterobacter agglomerans
138	24/2/019	Damietta	Common redshank (Tringatotanus)	healthy	-	-	O125 : H21	Proteus mirabilis
139	24/2/019	Damietta	Common redshank (Tringatotanus)	healthy	-	-	-	Hafnia species
140	24/2/019	Damietta	Common redshank (Tringatotanus)	healthy	-	Tsevie	-	Citrobacter freundii
141	24/2/019	Damietta	Common Quail (Coturnix coturnix)	healthy	-	Molade	-	Enterobacter aerogenes
142	24/2/019	Damietta	Common Quail (Coturnix coturnix)	healthy	-	Typhimurium	O128 : H2	Mixed Culture

143	24/2/019	Damietta	Common Quail (Coturnix coturnix)	healthy	-	Enteritidis	-	Klebsiella pneumoniae
144	24/2/019	Damietta	Ruff (PhilomachusPugnax)	healthy		Inganda	O55 : H7	Proteus mirabilis
145	24/2/019	Damietta	Ruff (PhilomachusPugnax)	healthy	-	-	-	Proteus mirabilis
146	24/2/019	Damietta	Spur-winged Plover (Vanellusspinosus)	healthy	-	Kentucky	-	Enterobacter aerogenes
147	3/2/019	Damietta	Northern Northern shoveler (Spatula clypeata)	healthy	-	Tsevie	-	Mixed Culture
148	3/2/019	Damietta	Northern Northern shoveler (Spatula clypeata)	healthy	-	Infantis	-	Hafnia species

Supplementary figure 1: Influenza viruses detection and subtyping using real time amd conventional PCR.

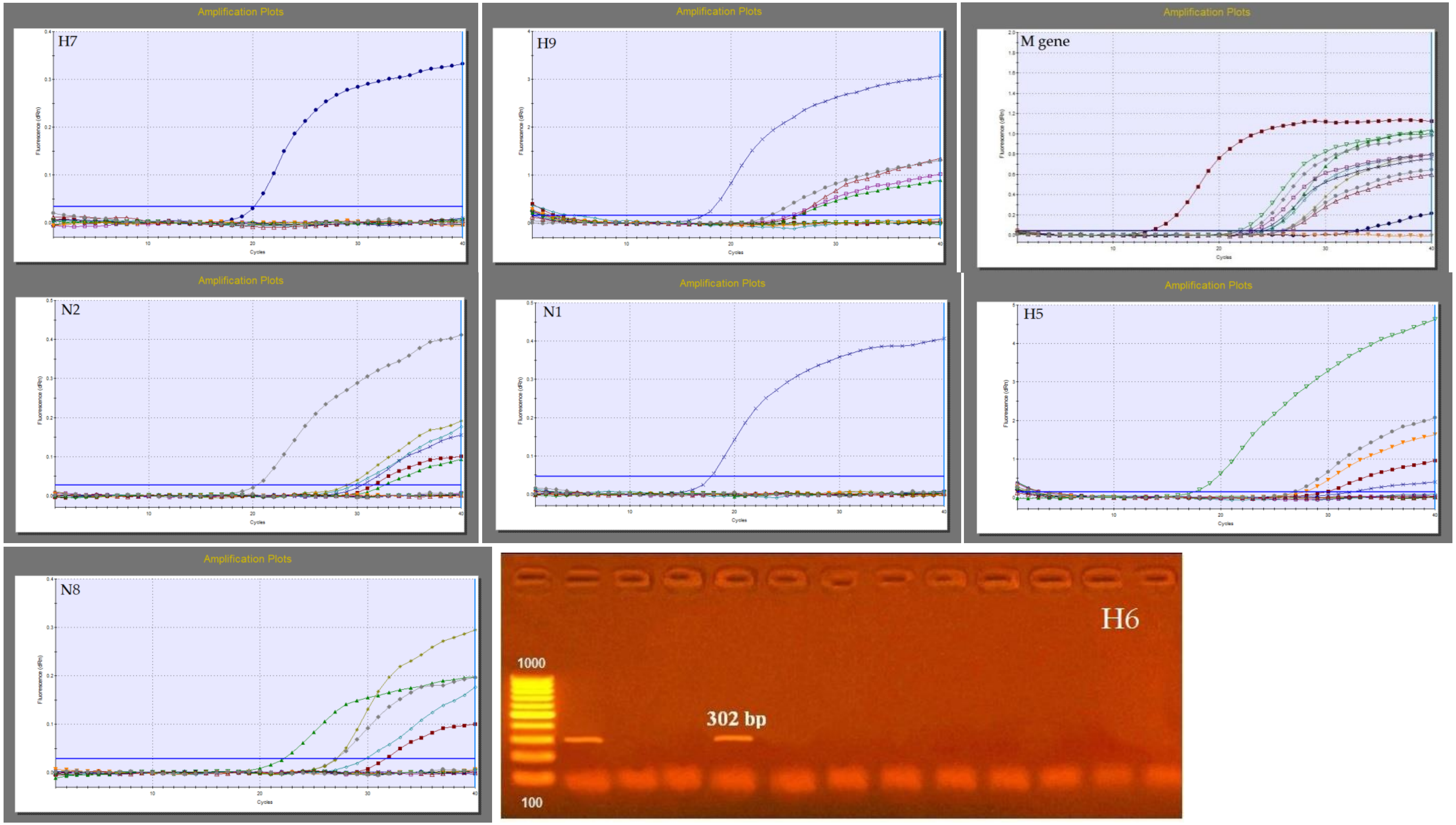

Supplement: Supplementary file 1 [file pathogens-09-00196-s001.pdf]
